# Narrative skill and testimonial accuracy in typically developing children and those with intellectual disabilities

**DOI:** 10.1002/acp.3427

**Published:** 2018-06-27

**Authors:** Deirdre A. Brown, Emma‐Jayne Brown, Charlie N. Lewis, Michael E. Lamb

**Affiliations:** ^1^ School of Psychology Victoria University of Wellington Wellington New Zealand; ^2^ Psychology Lancaster University Lancaster UK; ^3^ Psychology University of Cambridge Cambridge UK

**Keywords:** children, coherence, eyewitness testimony, intellectual disability, narrative quality

## Abstract

Children must describe maltreatment coherently for their testimony to be influential in court. We know little about how well children with intellectual disabilities (CWID) describe their experiences relative to typically developing (TD) children, despite CWID's vulnerability to maltreatment. We investigated children's reports of an experienced event and compared coherence in CWID (mild to moderate impairment: 7–11 years) with TD children matched for mental (4–10 years) or chronological age (7–11 years). All children included important markers of narrative coherence in their reports. Children with lower mental ages, particularly those with an intellectual disability, included fewer markers of narrative coherence in their reports than children with higher mental ages. Individual markers of narrative coherence, particularly recall of content, predicted accuracy of testimony and resistance to suggestion even when disability and mental age were taken into account. These findings highlight the importance of helping children to describe their experiences coherently.

## 1 INTRODUCTION

When children give testimony about maltreatment, the extent to which they can effectively convey their experiences influences whether their complaints are pursued in court and how credible they appear (Davis, Hoyano, Keenan, Maitland, & Morgan, 1999; Henry, Ridley, Perry, & Crane, 2011; Pipe, Orbach, Lamb, Abbott, & Stewart, 2013). Children's accounts are often the primary forms of evidence in cases of maltreatment, so listeners (including investigators and jury members) must be able to understand children's responses to questioning. If they are unable to make sense of children's accounts for any reason (i.e., ambiguities, lack of structure or organization, and incoherence), the credibility and impact of their evidence may be diminished (Davis et al., 1999; Westcott & Kynan, 2004). Narrative coherence also plays a role in how well children remember events and is associated with increases in the amount, accuracy, and retention of their reports of their experiences (Kleinknecht & Beike, 2004; Kulkofsky, Wang, & Ceci, 2008; Morris, Baker‐Ward, & Bauer, 2010; Qi, Van‐Kim, & Qingfang, 2015). Understanding how well children can convey their experiences is clearly important for evaluating any impact that their testimony may have on investigations and subsequent trials or decision‐making.

Very little research has examined the coherence of children's eyewitness accounts, despite the impact of (in) coherence on investigative and prosecutorial decisions. The limited evidence suggests that, as with other dimensions of eyewitness testimony (e.g., amount and accuracy), narrative skill is influenced by both individual (e.g., developmental level; Feltis, Powell, & Roberts, 2011) and interview (e.g., questioning strategy: Feltis, Powell, Snow, & Hughes‐Scholes, 2010) factors.

Narrative coherence has been studied in diverse ways, but most researchers emphasize the importance of contextual information, descriptions of the incident, the chronology, and evaluative statements which combine to help naïve listeners understand the speaker's experience (Reese et al., 2011). Some scholars have suggested that the various components of coherence emerge independently at different developmental stages and rely on different cognitive and social skills (Fivush, McDermott Sales, & Bohanek, 2008; Reese et al., 2011). For example, to contextualize an account adequately, the speaker must consider the listener's prior knowledge of the context and determine what additional information might help listeners understand what is being communicated. Such abilities rely on advanced social understanding (Carpendale & Lewis, 2004) as well as meta‐linguistic skills (Reese et al., 2011; Saywitz & Snyder, 1996). References to time and place appear to become stable in middle childhood but continue to develop into adolescence (Graffam Walker & Kenniston, 2013). To include information about chronology in their narratives, children must understand sequencing, temporal relations, and causal relationships between event components. Some of these understandings emerge early in development (e.g., preschoolers show an understanding of temporal sequencing), whereas others (e.g., causal reasoning) develop much later in childhood. Reese et al. (2011) showed that some early markers of narrative coherence are evident in very young (e.g., preschool) children's descriptions of events, but both within and across categories of coherence markers the complexity and number of narrative features increases with age.

In this study, we examine the extent to which narrative coherence is influenced by children's developmental level and cognitive ability. Very young children, and those with lower levels of cognitive function, may struggle to construct coherent accounts of their experiences for a number of reasons, including ineffective memory retrieval, difficulties with language and communication, and limited social understanding. Children's ability to recount their experiences coherently is also, in part, shaped by early interactional styles with parents and caregivers (e.g., Nelson, 2014). Children with intellectual disability (CWID) are less likely than their typically developing (TD) peers to be provided with the kinds of conversational scaffolding that would support the development of narrative skills (Agnew, Powell, & Snow, 2006; Hatton, 1998). Thus, given the cognitive and social challenges faced by CWID, which may equate to a level of functioning equivalent to that of much younger TD children, and limited opportunities for developing narrative skill, we might expect that they would describe events in a less coherent way than TD children. Examining the contributions of both developmental and cognitive levels of functioning to children's narrative skill may expand our understanding of how they come to develop an ability to describe their experiences effectively. It would also inform us about adults' strategies for supporting children who have not yet achieved proficiency, in contexts where the impact of their narratives may be critical to decision‐making (e.g., investigative interviews and court hearings).

Coherence scores are independent of the number of evidential details reported by children and also the accuracy and consistency of the details recounted. Indeed, a coherent account is not necessarily rich in detail nor composed of accurate information (Reese et al., 2011). Coherence relates to how children *structure* and *convey* their accounts to ensure a shared understanding with listeners of what occurred. Thus, a well‐organized or coherent narrative may be sparse in detail, and, conversely, an account that has a high level of detail may be poorly organized or incoherent. Field and laboratory‐based studies of narrative coherence in forensic contexts have tended to adopt a story grammar approach (see Kulkofsky et al., 2008; Reese et al., 2011, for examples of other approaches to coding coherence in autobiographical recall). According to the story grammar framework, successful narratives comprise six categories of information (Stein & Nezworski, 1978; see Table 1 for details of the categories), which provide information about the context, content, and characters associated with an event.

**Table 1:** Story grammar categories (Stein & Nezworski, 1978)

Category	Definition
Setting	Provides contextual details about the participants, social aspects, location and timing of the event
Initiating event	Describes how the event began
Internal response	Captures the emotions, cognitions and goals of the characters
Attempt	Describes the activities constituting the event
Consequence	Describes the outcome of the event
Reaction	Captures details about the feelings, thoughts, actions and consequences of the event

Research suggests that children may have difficulty constructing coherent accounts when recounting maltreatment. Westcott and Kynan (2004) evaluated the inclusion of story grammar components in forensic interview transcripts from TD 7‐ to 12‐year‐olds. Although all accounts (regardless of age) included some essential story components, they were often ambiguous, disordered, and incomplete. Free narrative accounts were most likely to contain important narrative information, but the components were often vague and the interviewers subsequently relied heavily on specific questions to elicit further detail. Nearly half of the accounts were disordered enough to impair the listener's ability to understand the evidence.

Westcott and Kynan (2004) suggested that a reliance on specific questioning was likely to increase the fragmented nature of children's accounts by interrupting the flow of children's recall, an observation that was supported by two field studies that examined the influence of questioning on children's coherence more directly. Snow, Powell, and Murfett (2009) and Feltis et al. (2010) found that open invitations were more likely to elicit story grammar details in forensic interview transcripts. Eliciting children's accounts with prompts that allow uninterrupted narrative responses is clearly important for enhancing the coherence of children's accounts. In the present study, interviewers followed an interview protocol shown to increase the use of open prompts and promote detailed responding from children (La Rooy et al., 2015). This allowed us to examine narrative coherence when an optimal questioning style was used.

The research described above highlights the challenges that TD children face when recounting their experiences in a forensic context, but what of children with cognitive impairments? Evaluations of children's coherence in an eyewitness context have almost exclusively considered typically developing participants, yet, as we outline below, CWID are a particularly vulnerable group of witnesses, with comparatively little research available to inform those who must elicit their testimony.

CWID are characterized by low levels of cognitive ability, alongside impairments in adaptive function or everyday living (American Psychiatric Association, 2013). CWID are underrepresented in all aspects of eyewitness testimony research, despite being particularly vulnerable to maltreatment. Rates of maltreatment are more than three times greater in CWID (31%) than in TD (9%) children (Sullivan & Knutson, 2000), and more severe types of sexual abuse have been demonstrated (Hershkowitz, Lamb, & Horowitz, 2007). Yet these children are less likely to have their complaints pursued in the criminal justice system (Reiter, Bryen, & Shachar, 2007; Sharp, 2001).

Interviewers are less likely to use open questioning with CWID than with TD children (Agnew et al., 2006; [Blinded for review]; Cederborg & Lamb, 2008). Thus, CWID are likely to be compromised, not only by their communicative and cognitive limitations but also by an interviewing style that is likely to diminish the coherence of the descriptions that these children provide. The goal of this study was to complement our existing knowledge of CWID's eyewitness ability by examining the extent to which they structure their recollections in a coherent manner.

Two studies have examined the narrative skills of CWID. Both suggested that their narratives were poorer in several ways than those of TD children. Murfett, Powell, and Snow (2008) found that CWID (9–12 years old) omitted more story‐telling components than did TD children (both mental‐age and chronological‐age matches) when describing witnessed interactions in response to open‐ended prompting. As a result, the clarity and comprehensibility of their accounts was reduced. Similarly, Gentle, Milne, Powell, and Sharman (2013) found that CWID (7–10 years) were less likely than TD children matched for chronological age to include important narrative components when describing a brief video clip watched the day before. These studies suggest that CWID may be less capable than TD children of organizing their narratives and including important elements to facilitate comprehension. However, directly experienced events are better remembered than those observed or heard about (Baker‐Ward, Hess, & Flanagan, 1990; Murachver, Pipe, Gordon, Owens, & Fivush, 1996; Pathman, Samson, Dugas, Cabeza, & Bauer, 2011), potentially providing a platform for greater coherence when recounting the experience, so it is important to examine narrative coherence when children are describing something that they were personally involved in.

In the current study, we examined data collected as part of a broader research program on CWID's eyewitness testimony (see Blinded for review) and focused specifically on the coherence of children's reports. We considered developmental differences in the coherence of children's testimony in two ways. Firstly, we examined the accounts of TD children across a wide age range (4–11 years), allowing for an examination of how coherence develops with age. Secondly, we included CWID with a range of mild or moderate levels of cognitive impairment (and therefore varying mental ages), thereby allowing us to consider development from the perspective of severity of intellectual disability.

We took a broad approach to measuring coherence by including story grammar elements (e.g., Gentle et al., 2013; Murfett et al., 2008; Westcott & Kynan, 2004) as well as information describing chronology, content, context and evaluation, which are markers of narrative coherence emphasized in linguistic and memory‐oriented studies (Kulkofsky et al., 2008; Reese et al., 2011). We examined children's recall of a personally experienced, interactive (rather than witnessed) event. This may provide a richer context for assessing narrative quality while enhancing the similarity between our research and the forensic context.

We examined recall of a staged event so we could also assess the accuracy of the children's accounts. Coherence is a predictor of the longevity of a memory, perhaps because it leads to stronger organization and consolidation of information (Morris et al., 2010). Thus, coherence may also assist children in preserving the accuracy of their accounts, and resisting suggestive questions, by virtue of supporting a stronger memory trace (Morris et al., 2010). It is important to determine whether a better story (i.e., a more coherent one) is also a more accurate one, because investigators and jury members appear to perceive the two dimensions of testimony as interchangeable.

We extended previous research by examining children's coherence when they were interviewed using the National Institute of Child Health and Human Development (NICHD) Investigative Interview Protocol (Orbach et al., 2000). Although Murfett et al. (2008) only included open‐ended questions, they limited the number of prompts used which may have also constrained the children's accounts. The NICHD Protocol includes a substantial preinterview phase, which includes a practice in episodic recall. Participants are asked to recall recent past events (e.g., their day until meeting with the interviewer) and are prompted to provide an elaborative account using the same kind of open‐ended questioning strategy during the subsequent target interview. Children are thus carefully prepared to provide detailed narrative accounts, which may influence reporting (e.g., Brown et al., 2013). As the protocol is a child‐centered approach, with flexibility in how the interview is constructed, transcripts are variably comprised of very broad open‐ended prompts and more focused follow‐up/cued recall questions. Westcott and Kynan (2004) showed that such questions may also elicit story grammar elements and so we did not restrict our analysis to the first spontaneous narratives provided by the children but instead considered how coherent their accounts were across the entirety of the interview. We examined the coherence of children's narratives after controlling for the overall number and proportions of different types of questions asked to eliminate the possibility that group differences reflected how children were asked about their experience rather than their ability to structure their accounts.

This study attempts to tease apart the influences of the child's mental age and the presence (or absence) of an ID. Several hypotheses derive from the literature. First, the number of narrative markers in children's accounts might increase simply as a factor of mental age (and irrespective of intellectual disability) (Morris et al., 2010; Reese et al., 2011). However, secondly, the presence of IDs (in comparison with TD) may be associated with fewer narrative markers (Gentle et al., 2013; Murfett et al., 2008). Further, based on studies showing poorer recall by children with moderate levels of impairment than typically developing children matched for mental age (Blinded for review), the more complex third hypothesis was that poorer narrative skills would be particularly evident in CWID with the lowest mental ages (those with moderate levels of ID), but that CWID with mild levels of impairment would be equivalent to their mental‐age matched groups. We did not make predictions about chronological age because, in this sample, some of the older children had intellectual difficulties. Fourthly, following Reese et al. (2011), we hypothesized that the various indicators of narrative coherence would be independent of each other. Following Kulkofsky et al. (2008), we expected, fifthly, to see an association between narrative coherence and the accuracy of children's accounts, at least in TD children. Lastly, we predicted that inclusions of fewer narrative components would be associated with susceptibility to suggestive questions posed at the end of the interview. While matching of ID children to TD controls is never straightforward we conducted analyses to allow us to compare intellectual status (TD vs ID) and mental age together, so that the above hypotheses could be compared.

## 2 METHOD

### 2.1 Participants

The data for this study were derived from interviews of the same children studied in previously published research (Blinded for review). Following approval from the University's Research Ethics Committee, 109 children (*n* = 109: 65 male) from four mainstream schools and five schools for children with intellectual disabilities were included in this study. There were 44 CWID and 65 TD children. All children gave verbal assent following written parental consent.

#### 2.1.1 Age

CA‐matched children ranged in age from 7 to 11 years, MA‐matched children ranged in age from 4 to 10 years, and CWID ranged from 7 to 11 years.

#### 2.1.2 Group allocation

Participants were initially categorized into CWID‐Mod, CWID‐Mild, MA, or CA groups based on prorated IQ scores from subtests (Picture Completion, Information, Block Design and Vocabulary) of the Wechsler Preschool and Primary Scale of Intelligence—Third Edition, UK version (WPPSI‐III UK) or the Wechsler Intelligence Scale for Children—Third Edition, UK version (WISC‐III UK), which were the most recent versions available at the time of data collection. Children included in the study were capable of basic verbal communication (minimal phrase‐based speech), confirmed in consultation with the children's teachers. Those with intellectual disabilities arising from organic syndromes (e.g., Williams or Down Syndrome) and those with diagnoses of autistic spectrum disorder were excluded, because specific social, behavioral, and information processing differences have been observed in these groups (Henry, Bettaney, & Carney, 2011). Consultation with children's teachers suggested that none of the children had any documented comorbid behavioral difficulties (e.g., ADHD and conduct disorder). Univariate analysis of prorated IQ scores for the groups showed a significant main effect of condition, *F* (4, 94) = 159.83, *P* < 0.001, η_p_^2^ = 0.87; Tukey tests (with the Tukey–Kramer adjustment here and below) indicated that CWID (Mod) had lower scores than CWID (Mild), and that both groups differed from the CA‐ and MA‐matched groups (all *P*_s_ < 0.001: see Table 2).

**Table 2:** Characteristics of the sample

	CWID (Mild)	CWID (Moderate)	MA Matched (Mild)	MA Matched (Moderate)	CA Matched
*n*	23	21	17	15	33 (23 unique)
*N* (male)	15	17	9	9	15
*N* (female)	8	4	8	7	18
Mean age in months (*SD*)	115.96 (14.83)	118.67 (13.35)	84.71 (11.73)	62.4 (8.31)	123.17 (15.29)
Mean Mental Age in months (*SD*)	83.17 (11.31)	62.71 (9.85)	87.12 (12.39)	64.67 (12.62)	128.61 (24.09)
Mean estimated IQ (*SD*)	67.70 (7.13)	48.81 (2.89)	101.00 (10.8)	101.0 (11.03)	104.96 (11.08)
Range of estimated IQ scores	56–76	44–53	87–118	88–118	84–124
Duration of interview in minutes (*SD*)	27.70 (8.01)	29.55 (6.13)	24.35 (4.8)	26.27 (6.96)	22.17 (6.69)

*Note*. CWID: children with intellectual disabilities.

#### 2.1.3 Matching samples

CWID were individually matched with TD children as closely as possible on the basis of gender and either MA or CA. CWID and TD children were recruited simultaneously. The mental age of all children was established from the tables provided in the Wechsler manuals. When MA estimates were not available from the Wechsler manuals because the children's ages fell in the crossover band between the two instruments and the severity of ID made the range of MA estimates provided by the WISC‐III UK insufficient, MA was estimated using the formula: IQ = (MA/CA) × 100. We were not always able to identify a close unique match for each child from within the TD cohort recruited; when there was no individual match, the closest match was chosen from among the children available, meaning that 10 TD children served as CA matches for two CWID participants. In comparisons with matched controls, these 10 children were excluded from specific group analyses. MA matches were paired with CWID from either the Mild or the Moderate CWID group to provide unique MA‐matched groups for each of the ID samples. The final sample for these calculations was: CWID‐Mild = 23; CWID‐Mod = 21; MA controls matched to CWID‐Mild = 17; MA controls matched to CWID‐Moderate = 15; CA matched = 23.

Characteristics of the sample are presented in Table 2. The two left hand columns provide the background details on sample size, CA, MA, and IQ in the two ID groups. The three remaining columns show the same information for the children who were closely matched in MA to each ID group and for the remaining children matched on CA.

#### 2.1.4 Age comparisons

A check was made to examine the success of the matching strategy. A univariate analysis of variance (ANOVA) showed a significant main effect of chronological age for group (*F*(4, 94) = 68.19, *P* < 0.001, η_p_^2^ = 0.74). Tukey tests showed, as expected, that children in the MA‐Moderate group were significantly younger than the MA‐Mild children who were younger than those in the other groups (all *P* < 0.001; see Table 2), who did not differ from each other. A univariate ANOVA showed a significant difference in the mental age of groups (*F*(4, 94) = 68.62, *P* < 0.001, η_p_^2^ = 0.74). Tukey tests showed that children in the CA group had higher mental ages than those in both the CWID‐Mild and their Matched MA group, who had higher mental ages than the CWID‐Moderate and their Matched MA group (all *P* < 0.01). Thus, the pairing of the each CWID subgroup with its comparison group in terms of MA, and the equivalence of the CA matches for the two levels of ID, was confirmed.

### 2.2 Procedure

#### 2.2.1 Event

The class‐based event was conducted in the children's schools and lasted approximately 40 min. Children were allocated to different teams of about six children, led by a research assistant, and participated in three activity stations about first aid and safety. At one station, children identified dangerous hazards depicted in large posters and discussed how the hazard might be overcome. At the second station, participants watched a video clip that showed a boy having a minor accident. The video demonstrated step‐by‐step care of minor cuts and abrasions. Children learned and then demonstrated how to take care of a simple cut by applying a novelty sticking plaster they had selected. At the third station, children learned how to tie a triangular bandage and practiced on each other under supervision. After this activity, the event leader took a photo of the children with their group leader. Part‐way through the event, a fourth research assistant interrupted the class and staged a brief argument about the equipment. After participating in the three activity stations, the children gathered as a large group and were reminded of the goal of each activity and received a small gift (novelty pencils). All children became highly engaged in the activities and attended to the interruption.

#### 2.2.2 Interview

The interviews were also conducted at school, 1 week after the event. One research assistant conducted the cognitive assessment and then acted as interviewer, with one of the others acting as a monitor, following typical procedures used in forensic interviews. Three research assistants conducted the interviews; no effect of interviewer on total amount of information reported was evident (*F*(2, 97 = 1.02). All interviewers completed a 2‐day training workshop in the use of the NICHD Interview Protocol. The workshop was delivered by the first author, who had previously been trained in the use of the protocol by its developers and using materials provided by them (Orbach et al., 2000). The workshop covered research in child development, the foundation and content of the protocol, discussion of transcript examples of good practice, and role plays of interviews with feedback. Interviews were regularly monitored by the first author to ensure adherence to the protocol. Interviewers participated in feedback sessions that included viewing the videotapes of their interviews, reviewing the transcripts, and refresher training, scheduled throughout the study. Two research assistants were present for each interview (one as interviewer and one as monitor) and provided additional feedback to each other after each interview to assist in maintaining fidelity and comparable performance.

Each interview began with rapport building, explanation of the “ground rules” (say “I don't know” as needed, do not guess, correct the interviewer if she made a mistake, tell the truth), and a practice in episodic memory recall (recall of the morning's events until meeting the interviewer). Focus was then shifted to the staged events using progressively informative prompts to orient the children to the information that was required. Prompts used throughout the interview were outlined in the NICHD Protocol. After the most *open prompts* were used (e.g., “tell me about that time”), additional recall‐based follow‐up questions might be *cued invitations* (e.g., “you mentioned you got to choose a plaster; tell me more about choosing the plaster”), or *direct questions* (e.g., “which plaster did you choose?”). Recognition‐based prompts were also used (e.g., yes/no or option‐posing questions) to clarify details. When the interviewer finished eliciting information, a short break was taken to allow the monitoring interviewer to suggest further prompts to clarify unclear or contradictory information. At the end of the interview, children were asked 16 suggestive questions. Half of the questions were open‐ended (“wh‐” questions,e.g., “where did you have a line drawn?”) and the remainder were closed (e.g., “did you have a line drawn on your finger?”). Within these categories, half were leading (asking about details that did occur, as in the open‐ended example above) and half were misleading (asking about details that were inconsistent with what occurred,e.g., “Where did your group leader cut you?”, or “did you have a line drawn on your knee?”). All interviews were transcribed verbatim from the video recordings, which included all interviewer and child utterances (including facilitative utterances such as “mmhmm”). Behavioral responses were described in full (e.g., children demonstrating how to tie a triangular bandage) and this information was included in the coding.

The lowest row in Table 2 presents the time taken to complete the interview. An ANOVA showed that the duration of the interviews (in minutes) was significantly different across the groups (*F*(4, 93) = 3.93, *P* = 0.005, η_p_^2^ = 0.15), with Tukey tests (*P* < 0.05) showing that the two CWID groups' interviews were longer than those of the CA matched children.

#### 2.2.3 Assessment of narrative coherence

Narrative coherence was assessed based on key story grammar elements, following Westcott and Kynan (2004) who adapted them from Stein and Nezworski (1978). Additionally, following Peterson and McCabe (1991) and Kulkofsky et al. (2008), we also coded for temporal features included in the children's reports. Only unique utterances were coded (repeated information was ignored). Information that was suggested or introduced by the interviewer (i.e., provided in the introductory or subsequent follow‐up prompts) was not coded.

The following categories were employed: Setting (Protagonist, Social, Temporal, Physical), Initiating event, Internal response, Attempt (descriptions of the activity stations, or the activity content), Consequence, Reaction, Simple temporal markers, and Complex temporal markers (see Table 3 for definitions and examples). *Total story grammar* elements were calculated as the sum of information reported in any of the Setting, Initiating event, Internal response, Attempt, Consequence, and Reaction categories. Each category was only awarded once within an utterance, to prevent confounding level of detail reported with the type of story grammar element the utterance relayed. To capture commonly identified aspects of narrative coherence (e.g., Reese et al., 2011), four measures were formed. A measure of *Chronology* comprised the sum of information coded as Setting‐Temporal, Simple temporal markers, and Complex temporal markers. *Content* was formed from the sum of information about the Initiating Event, Attempt, and Consequence. *Context* was formed from the sum of all Setting information, with the exception of Setting‐Temporal scores, which were included in the Chronology variable. Finally, a measure of *Evaluation* was formed from the sum of information describing Reaction and Internal Response.

**Table 3:** Narrative ability: Story‐telling components

Story telling component	Definition	Example of responses
Setting		
Protagonist	People at the event	“I think there was two girls and two boys”
Social	The hierarchy of the event	“the man, who was the leader of our group”
Physical	Where the event took place	“in the hall”
Temporal	When the event took place	“about 10 o'clock”
Initiating event	How the event began	“sat down. Charlie told us the groups we were going in”
Internal response	Emotions, cognitions, goals of the people	“She was nice, she was kind”, “we pretended it was a cut”
Attempt		
Activity Stations	Activities at the four stations constituting the health and safety event	“We looked at this picture”
Description	Description about the content of activities	“To see what was dangerous or not”
Consequence	What happened after the event	“We got a pencil”, “we went back to class”
Reaction	Feeling, thought, action, consequence from the event	“Now we know all about health and safety”
Simple temporal markers	Simple descriptors of aspects of chronology	“first, next, then, before, after”
Complex temporal markers	Temporal indicators of conditional states	“if, then, until, when”

#### 2.2.4 Accuracy

The accuracy of the details children reported was determined by reference to the event script, the video recording, and notes made by the research assistants at the time it was conducted. Accuracy was calculated as the proportion of all details that were correct (correct/total details). Accuracy in response to the suggestive questions was calculated as correct responses/number of suggestive questions asked.

#### 2.2.5 Coding

A subset of all transcripts (20%) was coded by an independent research assistant, blind to the hypotheses and design of the study. The kappa value was. 76, which reflects a substantial level of agreement (values were acceptable across each code, ranging from. 61–.80; Landis & Koch, 1977). The lead coder, who was blind to group membership, coded the remaining 80% of the sample, and recoded a subset (10%) to check for drift, which produced a kappa value of 0.85.

## 3 RESULTS

Table 4 presents the scores for the five measures of narrative coherence where the data are displayed in the same two ways as in Table 2, with the addition of the total sample in the left‐hand column. It shows that the children produced, on average, over 30 story grammar elements in their accounts and that references to chronology, the content of the event, and context were much more common than comments evaluating what happened. Table 4 allows comparison between the intellectual disability (ID) and the typically developing (TD) groups, as well as the data separated by severity of ID and the respective CA and MA matched groups.

**Table 4:** Mean (*SD*) number of markers of narrative coherence, total details included, and accuracy of children's reports shown, respectively, in the total sample, the TD and ID samples combined, the two CWID groups and matched groups, and the CA matched sample

	Total Sample	ID group	TD group	CWID‐ Mild	CWID‐Moderate	MA‐matched (Mild)	MA matched (Moderate)	CA‐matched
Total story grammar	32.27 (12.36)	27.36 (12.09)	35.58 (11.49)	34.96 (8.93)	19.05 (9.35)	37.53 (8.91)	28.00 (13.42)	40.39 (10.12)
Chronology	24.49 (16.35)	18.02 (14.60)	28.86 (16.12)	23.78 (13.80)	11.71 (13.00)	27.76 (14.90)	19.80 (16.15)	35.57 (17.00)
Content	10.87 (3.47)	9.61 (4.06)	11.72 (2.71)	11.74 (2.32)	7.29 (4.31)	12.41 (3.08)	9.93 (3.94)	12.35 (1.43)
Context	19.05 (8.62)	15.98 (8.57)	21.02 (8.06)	21.04 (7.16)	10.43 (6.31)	21.71 (6.58)	16.33 (8.71)	24.78 (7.44)
Evaluation	2.34 (2.12)	1.77 (1.38)	2.72 (2.43)	2.17 (1.34)	1.33 (1.32)	3.41 (2.72)	1.73 (1.89)	3.22 (2.63)
Total details	90.39 (34.50)	75.30 (32.26)	100.6 (31.88)	95.78 (24.27)	52.86 (24.05)	103.41 (30.96)	82.80 (35.80)	112.87 (28.59)
Total accuracy	.85 (.09)	.82 (.10)	.88 (.078)	.86 (.07)	.77 (.10)	.90 (.04)	.80 (.10)	.91 (.04)

*Note*. CWID: children with intellectual disabilities.

### 3.1 Statistical design

It was not possible to link each ID child with unique MA‐ and CA‐matched controls, so we analyzed the data in two ways. First, we examined CA and MA as continuous covariates. Almost half the sample had a formal diagnosis of intellectual disability so we looked at the effect of group membership (TD vs. ID) and examined group membership differences further by including the interaction of Group with CA and MA. We examined the measures of narrative coherence in a series of ANCOVAs, which also took into account the interviewer's contribution (the total number of questions asked of the child and the proportions of four of the five types of question used for each child [open invitations, cued invitations, direct prompts and option posing prompts: The few unscripted suggestive questions were omitted so that these proportions did not add up to one 1We did not include the proportions of suggestive questions asked because there were so few of these and to do so would have made the scores of all five measures add up to 1 and would have used up all the available degrees of freedom. When we ran the analyses with suggestive questions included there were no associations with the narrative skills of the children. Descriptions of the proportions of questions that comprised the interviews is analyzed in (citation blinded for review).]). These additional variables were included to rule out the possibility that the apparent narrative skill of the child was attributable to the interviewer's behavior. These analyses were followed by a series of planned comparisons (*t* tests) between each ID group with the TD subsample that was matched by MA. Finally, correlations examine how different aspects of narrative coherence relate to one another, and regressions to examine the relative effects of children's intellectual development on the one hand and their narrative skills on the other.

### 3.2 Markers of narrative coherence

First, we examined the total “number of story grammar elements” included in the children's reports. In the ANCOVA, we included the covariates (numbers of questions, proportion of open invitations, etc.) and main effects for CA, MA, and Group (ID vs. TD). We included the MA X Group interaction to test our predictions that developmental level would interact with ability (i.e., Group). None of the covariates or effects of CA was significant. There was a main effect of MA (*F*(1, 98) = 32.61, *P <* 0.001, η_p_^2^ = 0.23) and this was qualified by an MA X Group interaction (*F*(1, 100) = 4.67, *P* < 0.001, η_p_^2^ = 0.05). To unpack this interaction, separate models were run for each group. The effect of MA was stronger in the ID group (*F*(1, 36) = 22.41, *P* < 0.001, η_p_^2^ = 0.38) than for the TD children (*F*(1, 59) = 4.46, *P* = 0.04, η_p_^2^ = 0.05), but both remained significant—the more developmentally advanced the children (particularly in the ID group), the more story elements their narratives contained. This was confirmed by the planned comparisons, which showed that the CWID‐Mild children were no different from their matched MA group (*t*(38) = −.90, *P* = 0.37), whereas the CWID‐Moderate participants scored significantly lower than the MA matched subsample (*t*(34) = −2.36, *P* = 0.02, *d* = 0.77; see Table 4 for both sets of comparisons).

We next examined the inclusion of other markers of narrative coherence: Chronology, Content, Context, and Evaluation (see Table 4 for means). A MANCOVA with CA and MA as continuous variables revealed no effect of the total number of questions asked, proportions of each question type, or CA, but multivariate effects for Group (*F*(4, 97) = 2.83, *P* = 0.03, η_p_^2^ = 0.10), MA (*F*(4, 97) = 7.99, *P* < 0.001, η_p_^2^ = 0.25), and an MA X Group interaction (*F*(4, 97) = 3.16, *P* = 0.02, η_p_^2^ = 0.12). The univariate analyses showed a significant effect of Group on Content (*F*(1, 100) = 8.46, *P* = 0.001, η_p_^2^ = 0.08), significant MA differences on Chronology (*F*(1, 100) = 6.94, *P* = 0.01, η_p_^2^ = 0.07), Content (*F*(1, 100) = 17.20, *P* < 0.001, η_p_^2^ = 0.15) and Context (*F*(1, 100) = 29.56, *P* < 0.001, η_p_^2^ = 0.23) and a significant MA X Group interaction on Content (*F*(1, 100) = 6.01, *P* = 0.02, η_p_^2^ = 0.06) and Context (*F*(1, 100) = 4.67, *P* = 0.03, η_p_^2^ = 0.05).

To unpack the two interactions, we examined each intellectual disability group separately. For the Content of the children's narratives, the ANCOVA showed no effects for the TD children, but a strong effect of MA (*F*(1, 36) = 13.12, *P* = 0.001, η_p_^2^ = 0.27) in the ID sample. For Context, there were significant effects for MA in the TD group (*F*(1, 59) = 4.40, *P* = 0.04, η_p_^2^ = 0.07) and a stronger effect for ID children (*F*(1, 36) = 21.80, *P* < 0.001, η_p_^2^ = 0.38). When only the matched group was included, there were no differences from the CWID‐Mild sample on any measure (all *t*(34) values <1.8), but the CWID‐Moderate children reported significantly fewer elements about context than their MA matched group (*t*(34) = 2.36, *P* = 0.02, *d* = 0.78; see Table 4).

In summary, we found partial support for hypothesis one, and for hypotheses two and three, the narrative quality of the accounts was poorer in children with younger mental age and this was particularly the case for the children with intellectual disabilities. The five right hand columns in Table 4 show these effects in a categorical way, as the Mild ID and CA children represent the children in their respective groups (ID and TD) with a higher MA. CWID‐Mild produced accounts that were as coherent as those of TD children.

### 3.3 Relations among measures of narrative coherence in children's accounts

We conducted a series of Pearson's correlations between measures of Chronology, Content, Context, and Evaluation, first for the whole sample (Table 5, left hand panel), and then separately for each group of children (ID vs. TD: Table 5 center and right panels), to ascertain the extent to which the various aspects of narrative coherence were associated. Table 5 also presents correlations between the accuracy of information reported by children both in the interview and in response to the suggestive questions.

**Table 5:** Correlations among measures of narrative coherence, information provided, accuracy and resistance to suggestive questions

		CA	Chronology	Content	Evaluation	Context	Total Story Grammar	Accuracy	Suggestive question accuracy
Whole Sample	MA	.50**	.49**	.46**	.29**	.55**	.56**	.51**	.50**
	CA		.14	.13	.04	.15	.15	.21*	.11
	Chronology			.60**.	48**	.55**	‐	.36**	.46**
	Content				.38**	.65**	‐	.61**	.59**
	Evaluation					.48**	‐	.17	.25**
	Context						‐	.41**	.57**
	Total Story Grammar							.48**	.61**
	Accuracy								.44**
ID	MA	.35*	.41**	.60**	.03	.58**	.63**	.45**	.61**
	CA		.05	.29	.01	.03	.12	.09	.06
	Chronology			.62**	.40**	.47**	‐	.23	.46**
	Content				.19	.68**	‐	.58**	.64
	Evaluation					.18	‐	−.03	.12
	Context						‐‐	.34*	.58**
	Total Story Grammar							.43*	.64**
	Accuracy								.38*
TD	MA	.93**	.43**	.37**	.25*	.50**	.49**	.50**	.41**
	CA		.40**	.34**	.17	.43**	.42**	.57**	.40**
	Chronology			.54**	.48**	.51**	‐	.34**	.35**
	Content				.47**	.57**	‐	.57**	.42**
	Evaluation					.57**	‐	.18	.24**
	Context						‐	.34**	.58**
	Total Story Grammar							.44**	.47**
	Accuracy								.38**

* *P* < 0.05;

** *P* < 0.01.

In the entire sample, all measures of narrative coherence correlated significantly with each other (*P* < 0.001). In the TD and ID groups, the same pattern was evident except that chronology and evaluation in the ID group and chronology and evaluation in the TD group were not significant intercorrelated (see Table 5). Thus we did not, as predicted (hypothesis four), replicate Reese et al.'s (2011) finding that scores on the various dimensions were independent of one another. The correlations seem to show particularly strong links between chronology and the three other types of narrative skill as well as other associations.

In the total sample, overall accuracy and accuracy in response to the suggestive questions was related to Total Story Grammar scores and all of the individual narrative scores except for evaluation, where there was a nonsignificant trend (Table 5, left panel). Table 5 also shows that CA and MA were associated with both accuracy scores in the whole sample. When the two groups (TD, ID) were examined separately, MA related to accuracy in both groups, and CA was related to accuracy in the TD group. We next tested all the hypotheses, but particularly 5 and 6, by examining the relationship between the two measures of accuracy (right hand columns of each panel in Table 5) and the measures of narrative coherence. To control for CA, MA, and Group (ID vs. TD) as a dummy variable, we included these measures and the interaction between Group and MA and (as all the models were statistically significant) found the best‐fit standard regression model for each analysis. These are presented in Table 6.

**Table 6:** Summary of simple multiple regression analyses for variables predicting accuracy and resistance from the total and individual narrative scores

	B	S.E. B	β	*t*	Sig.
A. Accuracy (Predicted by Story grammar elements)					
MA	.003	.002	1.18	1.99	.05
CA	.001	.001	.22	1.24	.22
Group (TD vs. ID)	.17	.08	.94	2.12	.04
MA * Group	−.002	.001	−1.51	−1.8	.08
Total Story Grammar	.002	.001	.27	2.64	.009
Adj. *R* ^2^ = .32, *F*(5, 103) = 11.56, *P* < 0.001					
B. Accuracy (Predicted by Narrative coherence)					
MA	.001	.001	.26	1.44	.15
CA	.00	.001	.06	.50	.62
Group	.02	.03	.12	.82	.42
Chronology	.00	.001	−.10	−.82	.41
Content	.02	.003	.58	5.52	<.001
Context	−.001	.001	−.06	−.69	.49
Evaluation	−.003	.004	−.08	−.91	.37
Adj. *R* ^2^ = .43, *F*(7, 101) = 12.63, *P* < 0.001					
C. Resistance to suggestion (Predicted by Story grammar elements)					
i. ID Group MA	.006	.003	.39	2.35	.02
CA	−.002	.002	−.13	−1.06	.3
Total Story Grammar	.007	.003	.4	2.53	.02
Adj. *R* ^2^ = .45, *F*(3, 39) = 12.33, *P* < 0.001					
i. TD Group MA	.00	.002	−.07	‐ .21	.83
CA	.002	.002	.3	1.01.32	
Total Story Grammar	.006	.002	.39	3.11	.003
Adj. *R* ^2^ = .25, *F*(3, 61) = 8.06, *P* < 0.001					
d. Resistance to suggestion (Predicted by Narrative coherence)					
MA	.002	.001	.37	1.76	.08
CA	−.002	.001	−.19	1.00	.32
Group	−.03	.06	−.08	−.38	.70
Chronology	.001	.001	.07	.57	.57
Content	.02	.006	.35	3.06	.003
Context	.004	.003	.17	1.84	.07
Evaluation	−.007	.008	−.07	−.96	.34
Adj. *R* ^2^ = .41, *F*(7, 100) = 11.71, *P* < 0.001					

We first examined the Total Story Grammar scores, and then the four dimensions of narrative coherence for each dependent measure. Table 6 presents the four sets of analyses. Panel A presents the results for the analysis of whether Story Grammar scores predicted accuracy in the main interview. It shows that accuracy was predicted by MA (a near‐significant effect), Group membership (with TD children being more accurate), and Total Story Grammar.

When Total Story Grammar and all the control variables were loaded into the model predicting accuracy in response to suggestive questions, there was a significant MA X Group interaction (*t*(1) = 2.39, *P* = 0.02). Panel C in Table 6 thus presents the data for the two ID groups separately. It shows that Total Story Grammar contributed unique variance to the regression involving both TD and ID children, whereas, for the ID children, MA also contributed unique variance, thereby explaining the significance of the MA X ID interaction.

Panels B and D of Table 6 present the analyses conducted with the four types of narrative coherence included as predictors of the two accuracy measures. Both models fitted better when the interaction between MA and Group was included. In both analyses, the amount of Content information conveyed in the children's narratives predicted accuracy, whereas in response to the suggestive questions there was also a trend (*P* = 0.05) showing a positive relationship between MA and accuracy.

## 4 DISCUSSION

We examined the narrative coherence of the accounts CWID and TD children provided about a personally experienced event when they were interviewed using an interview protocol that encourages narrative reporting. Markers of narrative coherence were, in the main, frequently included in the children's reports, irrespective of group membership. As expected, markers of narrative coherence were positively related to mental age and negatively associated with intellectual disability. The picture was more complex when considering the CWID. Children with mild levels of intellectual disability provided similarly coherent accounts as typically developing children matched for mental age, but that those with moderate impairments were significantly less coherent in the context and content of their statements than typically developing children of similar mental age.

Our findings add to the existing literature regarding the coherence of CWID's narrative accounts of their experiences. Severity of ID (as measured by the interaction between mental age and ID) clearly affected the coherence of children's accounts, thereby supporting our suggestion that children with more severe levels of cognitive impairment may lack some of the cognitive and social skills needed to construct coherent narratives. The relatively impoverished quality of their accounts may also have reflected comorbid difficulties (e.g., language delays) that could influence how well they communicated their experiences. The inclusion of chronology, content, and context information was negatively associated with younger mental age, as was fewer story grammar elements. Thus, children with low mental ages (young TD children and older children with IDs and/or a low MA) may need particular help in constructing a coherent account of their experiences.

We found support for recent theories of the development of narrative ability across the lifespan (Reese et al., 2011), in the association between mental age and the total number of story grammar elements as well as the individual dimensions (with the exception of Evaluations). There were subtle differences, however, in the correlates of mental age in the ID and TD groups: In all cases, the effects of mental age were more evident in the ID groups (again with children with moderate intellectual disabilities performing worse than those with mild disabilities, and also worse than typically developing children of similar mental age). We suggest that, to develop Reese et al.’s (2011) model of narrative development fully, longitudinal work is needed to compare the developmental trajectories of ID and TD children. Children in all groups included more story grammar elements and other markers of narrative coherence (e.g., contextual details) than in previous studies (e.g., Murfett et al., 2008; Reese et al., 2011), perhaps because the types of events described differed (e.g., Fivush et al., 2008; Reese et al., 2011; Westcott & Kynan, 2004). The event we used was both substantial and structured, so the children may have included more elements than when describing self‐nominated autobiographical experiences (e.g., Fivush et al., 2008; Morris et al., 2010; Reese et al., 2011), brief witnessed events (e.g., Gentle et al., 2013; Murfett et al., 2008), or accounts of (typically repeated) alleged maltreatment (e.g., Feltis et al., 2010; Snow et al., 2009; Westcott & Kynan, 2004). Certainly, the forensic interview protocol used, with its emphasis on encouraging children to elaborate on previously disclosed information, may have provided optimal conditions for children to add to her or his narrative and thereby improve the coherence of the account.

Given the flexible questioning style encouraged by the interview protocol that we used, our statistical analyses took into account the possibility that interviewers may use different types of questions with different children. The influence of coherence on accuracy (in response to both recommended questioning and also highly suggestive questions) was evident even when we took question type into account. Our other work has showed that interviews are constructed differently with CWID and young TD children (Blinded for review), and yet here we see the capacity of these children to produce coherent descriptions of their experiences. Further research should explore how interviewers might elicit coherent as well as detailed narrative accounts from children, particularly those with young mental ages.

We had predicted that measures of narrative coherence would be independent of each other, as suggested by the model of Reese et al. (2011), but this was not the case. First, we saw correlations between all of the coherence variables, across the whole sample, and for the TD children, when groups were considered separately. Within the ID groups, content was also associated with chronology and context. The differences between our findings and those of Reese et al. (2011) could be explained by the use of different coding schemes (cf., Peterson, 2011). Indeed, using an alternative approach to coding, Kulkofsky et al. (2008) also reported significant positive correlations among all variables assessing aspects of narrative quality. Perhaps, the varying interview methods contributing to the data for the model of Reese et al. (2011) masked the competencies of younger children, revealed here when they were encouraged to provide a very detailed account of their experiences. Just as in other contexts where children learn from the adults around them (e.g., parent–child talk about the past, Nelson, 2014), we see in forensic interviews how children learn from unfamiliar adults even in the course of very brief interactions, with the style of interaction assuming great importance (Brown & Lamb, 2015, 2017). Testing this hypothesis is a key avenue for future research.

When examining associations comparing the relative influences of the total story elements, mental age, chronological age, and group membership on the accuracy of information children had reported, we found that only the total number of story grammar elements included predicted accuracy in both the main interview and suggestive questioning. When we examined specific markers of narrative coherence, only the ability to report the contents of the experienced event (how an episode started, what happened, and its consequences) helped to predict accuracy and resistance to suggestive questions. The positive association between narrative coherence and the accuracy of children's accounts is consistent with that observed by Kulkofsky et al. (2008). In the regression analyses, coherence, particularly the content of a narrative, predicted accuracy even when we controlled for group membership and different developmental levels.

The current findings not only challenge negative perceptions regarding the abilities of CWID to provide credible statements in forensic settings (see also Brown & Lewis, 2013; Henry, Ridley, et al., 2011; Nathanson & Platt, 2005) but also document greater narrative capacities than suggested by previous research (e.g., Gentle et al., 2013; Murfett et al., 2008). Importantly, they highlight the need for forensic interviewers to know more about the cognitive and communicative capacities of the children they are about to interview (Henry, Ridley, et al., 2011). Planning for the interview is important (Smith & Milne, 2011), and knowledge of the children's capacities and characteristics may help interviewers structure their interviews, select types of questions, and determine whether any additional support might be needed to facilitate the children's task.

Several topics for further research are apparent. Firstly, because researchers have employed different coding conventions when studying narrative quality in diverse (field and laboratory) contexts, and because of our modest sample size (especially for CWID), replication is essential. Secondly, we matched groups for age and gender, and examined the contribution of severity of ID to recall and coherence. An important additional consideration is the influence of language ability on narrative coherence, particularly in CWID, given the high comorbidity of developmental delay and language disorders. Finally, because children are often exposed to abuse on multiple occasions, and may be interviewed, either formally or informally (e.g., by family members) more than once, it is important to consider how narrative coherence is affected by the frequency with which target events were experienced (Feltis et al., 2011), and whether it develops or changes with repeated retellings (Reese et al., 2011).

As with other aspects of children's eyewitness testimony (e.g., amount recalled, accuracy of information), the coherence of children's accounts appears to be influenced by a range of factors relating to the children themselves, the nature of the event being narrated, and the way in which the children's accounts were elicited. Each of these sources of influence should be examined in the context of children's narrative ability to assist in elucidating factors that may enhance or reduce the quality of children's accounts. Our study contributes to this body of research by demonstrating previously unidentified competencies in young TD children and those with intellectual difficulties which may facilitate their access to the justice system when maltreatment has been alleged.
